# Hyperbaric oxygen therapy for thalamic pain syndrome: case report

**DOI:** 10.3389/fneur.2024.1364716

**Published:** 2024-03-13

**Authors:** John Benjamin Slade, Nathan Kwan, Peter Lennox, Russell Gray

**Affiliations:** David Grant USAF Medical Center, Travis Air Force Base, Fairfield, CA, United States

**Keywords:** thalamic pain syndrome, hyperbaric oxygen, case report, central post-stroke pain, post-stroke thalamic pain

## Abstract

Thalamic pain syndrome is a distressing type of central post-stroke pain (CPSP) that occurs in up to 10% of cases following a cerebrovascular accident, typically with a delayed onset of signs and symptoms, and is often chronic or even life-long. Thalamic pain syndrome, as is the case for other CPSPs, is difficult to treat, and the response is typically moderate at best. Central pain also occurs after vascular insults in parts of the CNS other than the thalamus. Only a few patients present with the classic “Dejerine and Roussy syndrome,” so the term CPSP is preferred for describing neuropathic pain after stroke. There are no pathognomonic features of this syndrome. The thalamus probably has a substantial role in some patients with central pain, either as a pain generator or by abnormal processing of ascending input. Long-term post-stroke pain disorders can reduce the quality of life, affect mood, sleep, and social functioning, and can lead to suicide. Hemi-body pain is common in patients with thalamic lesions. Hyperbaric oxygen has known physiologic and pharmacologic effects with documented benefits in brain-related hemorrhages, acute and chronic stroke, traumatic brain injury, mild cognitive impairment, neurodegenerative diseases, and neuroprotection, but has never been reported as a treatment for thalamic pain syndrome. A 55-year-old man with a history of migraines suffered a right thalamic lacunar infarction following a brain angiogram to investigate a suspected AVM found on prior imaging that resulted in immediate left-sided weakness and numbness, evolving to severe chronic pain and subsequent stiffness. Diagnosed with thalamic pain syndrome, multiple pharmacologic therapies provided only partial relief for a year after the stroke. The patient’s symptoms resolved and quality of life markedly improved with hyperbaric oxygen therapy, as assessed by multiple validated questionnaires, thus it may be a treatment option for thalamic pain syndrome.

## Introduction

This is the first known case report of the use of hyperbaric oxygen (HBO2) for the treatment of thalamic pain syndrome, also known as Dejerine–Roussy syndrome, a type of central post-stroke pain (CPSP), also known as post-stroke thalamic pain (PS-TP) (1).

Thalamic pain syndrome is a debilitating disease process that has limited treatment options.

A pure sensory stroke is a well-defined clinical entity with predominant hemisensory symptoms without other major neurological signs (2). The prognosis is typically poor. The character and severity of the pain will be persistent and often unchanging.

The prevalence of thalamic pain syndrome following a stroke is up to 10% of cases, and the onset of symptoms is often delayed, with the patient not experiencing significant pain until months or years after the stroke.

There are multiple reports of treatments for thalamic pain syndrome, including tricyclic antidepressants, anticonvulsants and opioid analgesics, intravenous morphine, lidocaine, and propofol (3), naloxone infusions (4), and Cilostazol (5). Proposed non-pharmacological treatments include neurostimulation (motor cortex, deep brain, and transcranial magnetic stimulation) for treatment-resistant cases of CPSP (3, 5), mirror therapy (6), ipsilateral stellate ganglion block (7), botulinum neurotoxin A injection (8), precentral gyrus stimulation, caloric vestibular stimulation, transcranial direct current stimulation, and bee venom acupuncture point injection (9).

HBO2 has been reported to be an effective treatment for multiple brain-related diagnoses, including TBI, acute stroke, neurodegenerative disease, and cognitive impairment. Proposed mechanisms of action include amelioration of oxidative stress, activating endogenous antioxidant activity, modulation of neuroinflammation, inhibiting apoptosis, stimulating pathways of neuroprotection, and modulating cerebral blood flow and brain metabolism (10).

In a randomized, prospective trial, the authors conclude that HBO2 during the degenerative (or acute post-stroke) stage could increase post-injury damage but that elevated oxygen during the regenerative stage would supply the energy needed for the innate brain repair processes. The study convincingly demonstrates that HBO2 can induce significant neurological improvement and that neuroplasticity can be operatively activated in chronic late-stage stroke patients long after the acute brain insult (11).

### Case description

This is the case of a 55-year-old man with thalamic pain syndrome. His past medical history includes asthma, CAD, diabetes type 2, hyperlipidemia, hypertension, and migraines. His past surgeries include gastric bypass, lumbar, and cervical fusions. A twin brother died in 2016 with end-stage renal disease and multiple strokes.

**Figure 1:**
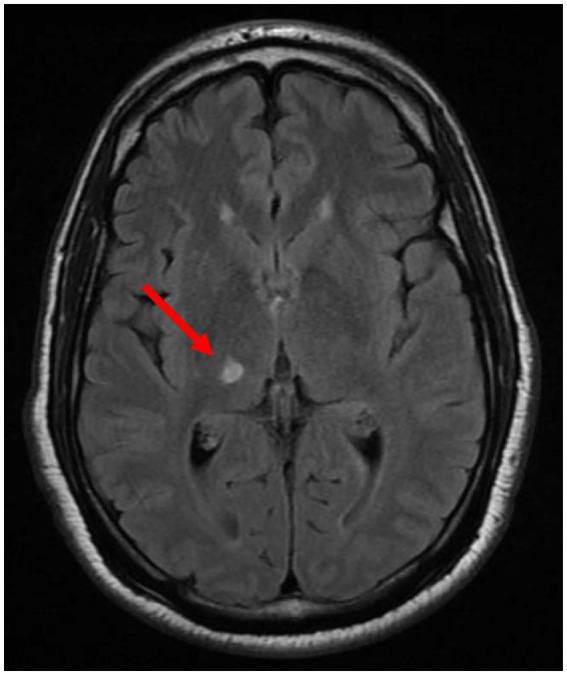
MRI January 2022 showing right thalamic lacunar infarction.

An MRI performed to evaluate migraines was significant for a focal prominence of the anterior portion of the inferior sagittal sinus, thought to represent a venous varix or focal tortuosity; it did not show arteriovenous shunting. The patient was referred to interventional radiology for further evaluation, with a cerebral angiogram conducted on 30 December 2021. The patient suffered an acute post-procedural thromboembolic right thalamic lacunar infarction, resulting in left-sided weakness and numbness including grip and foot drop. The patient was hospitalized and treated appropriately for an acute thromboembolic stroke with IV tenecteplase (TNK). Echo was normal without PFO, LDL 73, and A1c 5.8. The patient was placed on aspirin 325. A brain CT on 3 January 22 showed a 1 cm right thalamus lesion consistent with an acute infarct. Subsequent MRIs showed multifocal posterior circulation strokes, including the right MCA territory, notably the right thalamus (Figure 1).

The patient’s grip weakness and foot drop resolved completely in the subacute post-stroke stage, but numbness persisted. The patient developed severe left-sided burning hyperalgesia with an aftersensation described as a painful left hemi-body stiffness, which worsened 9 months post-cerebral vascular accident (post-CVA). In the ensuing year after the CVA, the patient experienced chronic, recurrent, frequent symptoms of intense acute left anterior chest burning pain that variably included the left face, followed predictably by a left hemi-body tightness/stiffness sensation that initially occurred 8–10 times/month, lasting an average of 2–3 days, rated 10/10 on the pain scale. Pain was paroxysmal burning or tingling, sometimes from the left ear distally.

The patient was evaluated in the ED over 10 times during that year for acute episodes and diagnosed with thalamic pain syndrome. Acute coronary syndrome was considered and ruled out.

Medical therapy included an anticonvulsant (gabapentin 300–600 gm t.i.d.), that variably provided partial relief approximately 1 h after administration, and a serotonin–noradrenaline reuptake inhibitor (SNRI, duloxetine 30–60 mg daily). The patient was initially treated with muscle relaxants, Methocarbamol, then baclofen (dose), and later cyclobenzaprine 10 mg t.i.d. PRN, all of which were subsequently discontinued due to marginal reported benefit. Pain medications included Norco 2/325 q6h PRN, Ibuprofen 600 mg q.i.d., PRN, and Tylenol. The patient reported symptomatic improvement with two short courses of Valium given during emergency room visits but was never prescribed for maintenance therapy.

The patient was managed by neurology as an inpatient, but not evaluated by outpatient neurology until 1 year post-CVA (Tables 1, 2).

**Table 1:** Outcome measurements and assessments.

	Initial/Baseline (completed retrospectively for January 2022)	After 57 HBO2 treatments (14 August 23)	After 71 HBO2 treatments (5 September 23)	After 80 HBO2 treatments (20 September 23)	Rating Scale
Pain Disability Index	39 Completely disabled	28 Severe disability	5 Mild disability	10 Mild disability	Increasing severity (0–60)
McGill Pain Questionnaire (SF-MPQ-2)	172 “Horrible”	89	60 “Mild”	6 No pain	Increasing severity (0–220)
Visual Analog Scale of Pain (DVPRS)	9.25	(No data)	2.75	1	Increasing Severity (0–10)
Beck Anxiety Inventory	49 Potentially concerning level of anxiety	3 Low anxiety	5 Low anxiety	4 Low anxiety	Low (0–21) Moderate (22–35) Potentially concerning (>/= 36)
Beck Depression Inventory	19 Borderline clinical depression	3 Normal	6 Normal	8 Normal	1–10 normal ups and downs 11–16 mild mood disturbance 17–20 borderline clinical depression 21–30 moderate depression 31–40 severe depression >40 extreme depression
EuroQol (Eq-5D-5L)	11/30	6/95	6/90	6/96	Level sum score (LSS)/VAS LSS: 0–5 no problems, 6–10 slight, 11–15 moderate, 16–20 severe, 21–25 extreme problems VAS: worst to best health (0–100)
Columbia Suicide Severity Rating Scale	0	0	0	0	Any score greater than 0 is important and may indicate the need for mental health intervention
Montgomery-Asberg Depression Rating Scale	3	5	No data	6	0–6 normal, symptoms absent 7–19 mild depression 20–34 moderate depression 35–60 severe depression

These validated tools are widely used and were selected from the outcome measurements suggested by Plow, et al. in a TPS RCT proposal (Trials. 2013. 14[241]).

**Table 2:** Timeline with relevant data from the episode of care.

29 Jun 2021 MRV Brain w/o contrast	4 mm venous outpouching from the inferior sagittal sinus may represent small arteriovenous fistula or arteriovenous malformation as it appears to have a component of arterial flow. Definitive evaluation of this structure can be obtained with catheter angiography.
30 December 2021 Cerebral angiogram	Acute CVA after femoral catheter removal complicated by post-procedural thromboembolic stroke with residual left-sided paresthesias and weakness.
4 January 2022	Admitted for suspected ACS, history of recurrent typical angina with undetectable cardiac troponin I levels on admission. Cardiac catheterization showed mild, non-obstructive coronary artery disease with 10–20% mid RCA stenosis, and 20–30% stenosis in the mid-distal RCA. Medical therapy was optimized.
10 January 2022	Seen in the ED for left-sided chest pain. CT head, MRI brain.
3 January 2023	Initial HBO2 consult, pain 10/10.
30 January 2023 (HBO2 #16)	Pain 4–6/10 -decreased duration of daily episodes. Decreased intensity, less stiffness.
10 February 2023 (#22)	Decreased severity and duration of flares.
16 February 2023	Continue gabapentin, duloxetine, stop Tylenol.
28 February–10 April 2023 (#33)	Break for a trip, 41 days.
10 April 2023	Return from trip, some recurrent episodes but not as severe as a baseline.
13 April 2023 (#36)	No left thorax burning paresthesia since December 2022, continued left-sided stiffness, less intense.
2 May 2023 (#47)	1, 2 May – worse stiffness, some relief with Tylenol, shorter duration than baseline.
15 May–14 Aug 2023 (#56)	Trip, no HBO2 for 90 days.
14 August 2023 (#57)	Return from trip, incremental loss of prior benefit.
18 September 2023 (#80)	Last planned HBO2 treatment, continued symptomatic improvement.
18 September–17 October 2023	Trip of 28 days.
17 October 2023	Decision to resume HBO2 with a plan for 20 treatments or to the plateau or resolution of symptoms.
15 November 2023 (#100)	Last HBO2 treatment, complete symptoms resolution, continues gabapentin, and duloxetine.

ED: 3–4, 10–11, 15, 18, 25 January 22, 7 September 2022, 19 December 2022, 1 November 2023.

Admit: 4, 11, 16, 25–28 January 2022, 19–20 December 2022.

### Diagnostic assessment

Criteria for the diagnosis of CPSP include pain in an area of the body corresponding to the CNS lesion, a history suggestive of stroke and pain onset at or after stroke onset, confirmation of the CNS lesion by imaging, and other causes of pain excluded or considered highly unlikely (3).

Diagnosis of PS-TP based on contralateral burning pain and spasticity after an imaging-demonstrated right thalamic ischemic stroke lesion that occurred at the time of a cerebral angiogram performed for a migraine workup, with associated showering of emboli.

One year post-CVA, the patient’s symptoms remained unabated, with partial, temporary relief from gabapentin 300–600 mg TID, duloxetine 60 mg daily, a muscle relaxant (baclofen, then Flexeril 10 mg TID PRN), and Tylenol. At the initiation of HBO2 treatments, the patient described at least daily pain episodes lasting approximately 1 h that began with a burning sensation in the left anterior chest, progressing to cramp-like pain involving his entire left side. The pain was consistently triggered by airplane flights, with no other obvious aggravating or relieving factors. It variably kept the patient awake at night. HBO2 treatments were administered at 2 atmospheres absolute (ATA) for 90 min each, the protocol routinely used at our multiplace hyperbaric chamber facility for non-emergent diagnoses and used by our group in prior research protocols for the treatment of traumatic brain injury. The initial plan was for 40 treatments based on the report in post-stroke patients to be effective for inducing late neuroplasticity (11) and that showed improvements in NIHSS, activities of daily living (ADL), and quality of life (QoL). However, due to family circumstances and personal obligations, the patient had to take three breaks in the therapy (41, 90, and 28 days), resulting in a treatment course of 100 HBO2 sessions over 11+ months, extended primarily due to ongoing progressive improvement.

The patient continued gabapentin 300–600 mg TID throughout the course of HBO2, required less Tylenol (initially multiple times daily to several times/week), replaced baclofen with Flexeril, and eventually stopped both.

After the initial 10 HBO2 treatments, the patient noted significantly reduced pain during the acute episodes, rated at 4–6/10 compared to his baseline of 10/10. The patient also noted the reduced duration of the acute episodes, lasting hours versus days, and decreased intensity of the left arm and leg stiffness that followed each pain episode. The frequency decreased to only two episodes in the first 3 weeks of HBO2 therapy. After 33 treatments, there was a 41-day gap in therapy due to a planned trip. On resuming HBO2, there was a slight loss of prior improvements, and at treatment #34, the patient had one of his worst pain flare episodes with severe left hemi-body pain and usual post-pain stiffness. The patient continued taking gabapentin 300 mg TID (earlier was 600 mg TID), rare use of Tylenol (earlier was 325 mg TID), duloxetine 30–60 mg/day, and Flexeril.

After 37 treatments, the patient was no longer having acute exacerbations and was only experiencing baseline hemi-body stiffness with no pain. The patient continued with gabapentin and duloxetine with the rare use of Tylenol.

After 56 treatments, there was a 90-day break in HBO2 therapy for another trip. On return, there was again a slight loss of prior improvements, and the decision was made to continue with a total of 80 treatments, at which time the patient had planned a trip lasting 28 days. Notably, the patient consistently had minor flares in symptoms for 2–3 days after airline flights. After the 28-day break, the patient felt that he was continuing to improve, and was given a final of 20 HBO2 sessions, for a total of 100 sessions over a period of 11 months. At the end of the 100 treatments, the patient was completely asymptomatic. He continued with gabapentin at 300 mg TID and duloxetine at 30 mg/day.

Considering that the patient was seen multiple times by various providers during the 11 months between the CVA and HBO2 and had stable, severe symptoms (no significant or sustained improvement), he was encouraged to maintain a stable medication regimen to best assess his response to HBO2 therapy.

## Discussion

Thalamic pain was first described by Dejerine and Roussy as, “… severe, persistent, paroxysmal, often intolerable, pains on the hemiplegic side, not yielding to any analgesic treatment” (12). Thalamic pain is characterized by constant or intermittent pain in the hemi-body, contralateral to the thalamic lesion, and is a severe and treatment-resistant type of CPSP that occurs after a cerebrovascular lesion results in thalamic stroke.

Treatment remains difficult, and the prognosis is typically poor. Possible pathogenesis includes central imbalance, central disinhibition, central sensitization, other thalamic adaptive changes, and local inflammatory responses (1).

PS-TP may develop immediately after a stroke, as in this case report. Up to 40% occur within a month of the stroke, over 40% have symptoms between 1 and 12 months, and sometimes develop 1 to 6 years post-stroke (2, 13, 14).

Right-sided stroke lesions were more commonly associated with the development of PS-TP than left-sided lesions (15).

In a series of patients with thalamic infarcts, only lesions in the ventral posterior part of the thalamus caused CPSP (16).

CPSP can develop after both hemorrhagic and ischemic lesions of the CNS. A lesion in the CNS results in anatomical, neurochemical, excitotoxic, and inflammatory changes, all of which might trigger an increase in neuronal excitability (17) that might lead to chronic pain.

### Treatments for thalamic pain syndrome

Treatment of CPSP is usually a combination of several drugs. Commonly recommended first and second lines of pharmacological therapies include traditional medications, e.g., tricyclic antidepressants, SSRIs, SNRIs, anticonvulsants, and opioid analgesics. Anticonvulsants have several mechanisms of action, including reducing neuronal hyperexcitability (3).

Non-pharmacological interventions, such as transcranial magnetic or direct current brain stimulations, vestibular caloric stimulation, epidural motor cortex stimulation (MCS), and deep brain stimulation (DBS), were effective in some case studies and can be recommended in the management of therapy-resistant PS-TP (1). Intracranial neurostimulation for pain relief is most frequently delivered by stimulating the motor cortex, the sensory thalamus, or the periaqueductal and periventricular gray matter. The stimulation of these sites through MCS and DBS has proven effective for treating a number of neuropathic and nociceptive pain states that are not responsive or amenable to other therapies or types of neurostimulation (18).

Recent data suggest that the therapy may have value for treating post-stroke pain, central pain syndromes, and peripheral deafferentation pain (19, 20). An “insertion effect” has been reported, in which the placement of DBS leads provides pain relief even when the leads are not activated (21).

Cilostazol, a selective and potent inhibitor of phosphodiesterase (PDE) 3A, likely increases the level of cAMP and has been reported to be effective (5).

Mexiletine, an orally active antiarrhythmic agent, produced improvement in eight of nine thalamic pain syndrome patients over 4 weeks (22).

In a case report, multidrug-resistant thalamic pain was successfully alleviated by modulating the contralateral thalamic pathway with ipsilateral stellate ganglion block (7).

A patient who developed CPSP 5 years after a right lenticular-capsular thalamic stroke was treated with 2 weeks of mirror therapy with a reduction in pain that was maintained at the 1-year follow-up (6).

Two cases of thalamic syndrome were treated with weekly, increasing doses of naloxone infusions successfully relieved pain for up to 6 months (4).

There is a growing body of evidence showing benefits with HBO2 in post-stroke patients, including post-stroke memory impairment (23), cognitive impairment (24), traumatic brain injury (25, 26), and chronic neuropathic pain (27).

A prospective, randomized, controlled trial of 74 patients, all of whom suffered a stroke 6–36 months prior to treatment with HBO2. The treatment protocol was 40 sessions (5 days/week) at 2 ATA. The authors concluded that HBO2 can lead to significant neurological improvements in stroke patients, even in chronic late stages. Improvements were measured by neurologic evaluation with NIHSS, brain functional imaging (SPECT), and QoL evaluations. The improvements support the idea that neuroplasticity can be activated months to years after stroke with proper stimulation, such as HBO2. This and other studies reveal that many aspects of the brain remain plastic even in adulthood (11). HBO2 can initiate cellular and vascular repair mechanisms and improve cerebral vascular flow. At the cellular level, HBO2 can improve neuronal and glial cell mitochondrial function, improve blood–brain barrier and inflammatory reactions, reduce apoptosis, alleviate oxidative stress, increase levels of neurotrophins, and nitric oxide, and upregulate axon guidance agents. HBO2 may promote the neurogenesis of endogenous neural stem cells (28).

In a review of four studies, the authors concluded that HBO2 is effective for ischemic strokes (26).

HBO2 can exert neuroprotective effects through multiple pathways in acute ischemic stroke (AIS), with consequent salvage of neurological function.

The three phases of potential HBO2 benefit in ischemic stroke are pretreatment, early (limited to 12 h from the time of onset of AIS), and recovery from neurological damage in the chronic phase (29).

In a large retrospective 2020 study including chronic stage post-stroke patients at least 3 months post-injury (median 1.5 ± 3.3-year post-stroke), HBO2 was found to induce significant improvements in all cognitive function domains. In 50 patients (30.86%), the stroke was subcortical. There were no significant differences in HBO2 effects on subcortical strokes compared to cortical strokes (30).

In a clinical study, six patients 1–2 years post-stroke with a stable baseline were treated with HBO2 at 2 ATA X for 60 min for a total of 40 treatments over a 12-week period. The authors reported improvements in cognition, gait velocity, upper extremity mobility, sleep, and overall recovery that were maintained for up to 3 months (31).

A review of HBO2 in acute stroke discussed physiological effects that include ameliorating AIS-induced brain tissue hypoxia, stabilizing the blood–brain barrier, decreasing intracranial pressure, and relieving cerebral edema (32).

HBO2 alleviates neuroinflammation through effects that include reduced inflammatory enzymes and inhibition of neutrophil infiltration and matrix metalloproteinases (MMP-9).

HBO2 can inhibit apoptosis and have neuroprotective effects. Mechanisms include increased expression of neurotrophic and nerve growth factors, mobilization and migration of pluripotent mesenchymal stem cells, proliferation of astrocytes, inhibition of the secretion of microglial cells, and improving outcomes for neurological and motor functions.

The timing of HBO2 in AIS may be an important factor in efficacy. Studies suggest that the ideally, HBO2 should be initiated within the first 3-5 hours post ischemic stroke (33, 34) and has been shown to be beneficial in the chronic phase (11).

HBO2 was shown in a retrospective study of 162 patients to induce significant improvements in all cognitive domains, even in the post-stroke late chronic stage. There were no significant differences in the HBO2 effect on cortical strokes compared to subcortical strokes. Participants were treated at 2 ATA for 90 min (with 5-min air breaks every 20 min) for 40–60 sessions. The median time from stroke to HBO2 was 1.5 ± 3.3 years. Neuroplasticity is induced by two main physiological effects: increasing tissue oxygenation and repeated oxygen level fluctuations, which increase HIF-1a. This, in turn, triggers regenerative processes in the metabolically injured brain areas (30).

HBO2 can improve cognitive functions in patients with mild cognitive impairment. Elevated humanin levels (mitochondrion-derived neuroprotective peptide) were reported in vascular dementia patients treated with HBO2. Maintenance of HBO2 will likely be needed for progressive neurodegenerative diseases (24).

Mechanisms of the neuroprotective effects of HBO2 in stroke patients include stabilizing the blood–brain barrier, improving metabolism, reducing brain edema, alleviating post-stroke neuroinflammation, inhibiting post-stroke apoptosis, improving anoxic area microcirculation, and suppressing ischemia–reperfusion injury (35).

The fact that the patient consistently experienced exacerbations of symptoms during and for 1–2 days after each airline flight strongly suggests that the neurological injury is sensitive to hypoxia, hypobaric pressure, or both. This same observation has been made in other clinical scenarios and was the basis for an experiment on the effect of commercial airline cabin pressure/hypoxic exposures incurred on evacuation flights of injured U.S. military members from Iraq. That experiment in acute brain-injured animals demonstrated amplification of the acute brain injury by hypobaric exposure to 8,000 feet altitude despite the correction of hypoxia (36). The patient’s sensitivity to hypobaric exposure at cabin altitude suggests that the opposite, exposure to increased pressure achieved with HBO2 therapy, is consistent with the observed clinical improvements.

### Study limitations

Baseline assessments were retrospective, based on the patient and his spouse’s recollection of symptoms during the year prior to initiation of HBO2 therapy.

It is unknown whether the patient would have responded differently without the interruptions in the HBO2 therapy.

A definite limitation in the case report is the short (1 month) follow-up. Fortuitously, the symptomatic responses noted during the multiple patient-imposed breaks in therapy, might offer some insight, and imply that the patient could require periodic HBO2 to maintain benefit, as has been suggested in the HBO2 treatment of other brain pathologies.

Questionnaires were completed for only 80 out of the 100 treatments. However, the patient was entirely asymptomatic at the end of HBO2 therapy and had no adverse effects or complications from HBO2 treatments.

### Study strengths

Multiple other pharmacologic and non-pharmacologic therapies have reported benefits in thalamic pain syndrome, but none have been attempted in this patient. There were no significant changes in the pharmacologic treatments during the study except for a reduction in pain medications. This allowed for clinical improvements to be attributed to the HBO2 intervention.

Reports of HBO2 mechanisms of action and efficacy in stroke and other brain pathologies support the clinical benefit realized in this case report and are consistent with a regenerative improvement in the chronic stages (1-year post-CVA in this case). HBO2 therapy is a potential treatment option for thalamic pain syndrome. The specific mechanisms of HBO2 therapy need to be further studied and explored.

### Patient perspective

I am glad that my wife suggested trying the HBO2 therapy. My symptoms started at the time of the cerebral angiogram (December 2021) but became most intense approximately 9 months later (September 2022), with only partial relief from medications. With each pain episode, I worried whether it would get worse. The year of HBO2 really relaxed my body, and it is the best I have felt since September 2022. The symptoms improved with each new course of HBO2. Overall, the entire year of HBO2 treatments was the best I have felt since onset; the episodes were less painful, shorter, and did not keep me from doing what I wanted to do.

## Data availability statement

The original contributions presented in the study are included in the article/supplementary material, further inquiries can be directed to the corresponding author.

## Ethics statement

Written informed consent was obtained from the individual(s) for the publication of any potentially identifiable images or data included in this article.

## Author contributions

JS: Writing – original draft, Writing – review & editing. NK: Writing – original draft. PL: Writing – original draft, Writing – review & editing. RG: Writing – review & editing.
